# Diseases of the Ear

**Published:** 1860-05

**Authors:** 


*Diseases of the Ear.* By J. TOYNBEE, F.R.S. Republished by Blanchard & Lea. Philadelphia: 1860.

We have carefully read this book through. It has the merit of being founded on the large experience furnished by dissections of over 2,000 cases. The disorders of this complicated and exquisitely delicate organ the author has judiciously grouped into anatomical divisions: diseases of the meatus and its canal; of the tympanum and its cavity; of the ossicula and labyrinth; caries and necrosis of the mastoid cells, and adjacent bone, reaching into the skull.

Injections are a favorite remedy of the author, and pervade throughout the work every variety of cases. At page 108, it is remarked: "Here, perhaps, I may be excused a few words on the subject so frequently adverted to, as the danger of stopping a discharge of the ear. * * * * Where strong injections have been employed, the symptoms that follow are not dependent upon the cessation of the discharge, but upon the inflammation caused by the irritant." Doubtless the injection can excite the existing chronic into acute inflammation; but, even if no inflammation be produced, the cessation of the discharge is too often merely the cessation of its *exit*, not of its formation; this goes on, though none escapes, as a *discharge*, pressing in all directions in search of an outlet, until it works through the thin leaf of bone that separates the seat of disorder from the cavity of the skull; *now* producing inflammation of the brain, almost always followed in a few days by death. Four such cases we have seen in adults, a few days previously in good health; the injection causing swelling and closure of the orifice that gave passage to the matter behind, arresting the discharge, but not its secretion. We must, in justice to the author, say, that in Chapter XII. he cautions against the indiscriminate use of *irritating* injections, lest they confine the pus, and cites many interesting cases wherein the roof of the tympanum and mastoid cells became pierced, the brain affected, speedily followed by death. We have thought proper to make the foregoing remarks, having too often noticed the *matter-of-course* prescription of injections by general practitioners, who have not made diseases of the ear a study—know little about the ear; and to those we strongly recommend this work, as filling up a vacuum that is left void in systems of surgery.

The typography and general getting up of the book deserve all praise; but we much regret that the wood-cuts, while accurate as to drawing, are completely spoiled by the deep shading; so much so, in most of them, as to defy the eye to make out what they are designed to illustrate.

